# Obesity and Insulin Resistance Are the Central Issues in Prevention of and Care for Comorbidities

**DOI:** 10.3390/healthcare3020408

**Published:** 2015-06-04

**Authors:** Elisabeth Govers

**Affiliations:** Amstelring Foundation for Primary Care, The Dutch Dietitians Knowledge Centre for Overweight and Obesity (KDOO), Amsterdam 1065AC, The Netherlands; E-Mail: e.govers112@upcmail.nl; Tel.: +31-643890145

**Keywords:** obesity, insulin resistance, metabolic syndrome, comorbidities, treatment, dietitian

## Abstract

For a long time the assumption has been that, although weight reduction was necessary and desirable, comorbidities were far more important and needed treatment even if weight loss was not a treatment goal, preferably with medication. This controversy leads to postponement of treatment, and later on causes very intensive medical treatment, thus, raising the health care costs to unacceptable levels, leading to the medicalization of individuals, and a declining of the responsibility of patients for their health, leaving the question of when to regard their own weight as a problem that should be dealt with up to individuals. The central problem is insulin resistance, which leads to a cascade of health problems. This condition should be diagnosed in primary practice and obesity clinics to ensure a better, tailor-made treatment for patients. Treatment should start at the earliest stage possible, when comorbidities are still reversible and includes a personalized dietary advice and counseling, preferably by a dietitian, to tackle insulin resistance. An exercise program is part of the treatment.

## 1. Introduction

For a long time the assumption has been that, although weight reduction was necessary and desirable, comorbidities were far more important and needed treatment, preferably with medication, even if weight loss was not a treatment goal. In several countries overweight is not even regarded as a disease, but as a condition, whereas, e.g., hypertension, dyslipidemia, and type 2 diabetes are considered as real diseases. This controversy leads to postponement of treatment, and, later on, causes very intensive medical treatment, thus, raising the health care costs to unacceptable levels, leading to the medicalization of individuals, and a declining of the responsibility of patients for their own health, leaving it up to individuals when to regard their own weight as a problem that should be dealt with. In this article I will explain why we need a shift of paradigm regarding the relationship between body weight and comorbidities.

## 2. Insulin Resistance

Extensive research has been carried out to reveal the mechanisms that cause insulin resistance [1,2,3,4,5], and others have continued by examining the relationship between obesity, cardiovascular disease, hypercoagulability, type 2 diabetes, non-alcoholic fatty liver disease, and insulin resistance [6,7,8,9,10,11]. Genome-wide studies found 97 BMI-associated loci, suggesting a role of the central nervous system in developing obesity. For instance, synaptic function, glutamate signaling, insulin secretion, energy metabolism, and adipogenesis may be genetically determined [12]. Genes also determine fat distribution to a great extent [13]. Glucose-clamp studies showed that comorbidities are caused by the presence of combined insulin resistance and hyperinsulinaemia, deriving from the inflamed adipose tissue, which is characterized by increased monocyte infiltration and cytokine production [8,14], Insulin resistance is the result of a long-term process that is encountered by chronic energetic overfeeding, when an abundance of glucose and saturated fat enter the cell, leading to Endoplasmic Reticulum (ER) stress; a low grade flammation process and hypoxia [1,14,15]. In short, extensive fat accumulation, usually due to overfeeding, overfills the present subcutaneous fat cells, and leads to fat accumulation in the abdomen, the visceral fat, muscles, and liver. The adipocytes in the visceral fat start to produce many adipokines, which alter different metabolic processes: serum lipids change (HDL cholesterol goes down, LDL cholesterol and triglycerides go up), blood pressure rises, purine levels rise, estrogen levels rise, testosterone levels go down, the thyroid gland may start to dysfunction, and the production of insulin increases to twenty times the normal level (hyperinsulinaemia). After a longer period of time, the pancreas fails in meeting insulin needs after meals, leading to impaired glucose tolerance, and finally to type 2 diabetes. Insulin resistance has also been linked to the prevalence of breast, prostate, and colon cancers. For prostate cancer it has been shown that hyperinsulinaemia acts on the liver to increase production of insulin-like growth factor-I (IGF-I), a factor known to stimulate tumor growth and block apoptosis [16]. Insulin resistance leads to over activity of mast cells in intestine, lung, and skin, causing allergic reactions. In children and adolescents, HOMA estimated insulin resistance values were significantly associated with positive skin tests and allergic asthma diagnosis. There was a strong relationship between a large waist circumference and pulmonic function [17]. Patients with mild stages of COPD often have obesity and insulin resistance. Patients with COPD and metabolic syndrome have increased risk of morbidity and mortality due to cardiovascular disease [18]. Growing evidence supports the concept that insulin resistance is important in the pathogenesis of cognitive impairment and neurodegeneration. Insulin plays a profound role in cognitive function. Impaired insulin signaling in the advancement of cognitive dysfunction is relevant to the pathophysiologic mechanisms of cognitive impairment and the risk of developing dementia [19,20]. The relationship between sleep apnea and metabolic syndrome is well known [21].

Finally, recent studies have shown that duodenal dysfunction in obese persons is a consequence of their weight. Pattern recognition receptors, as well as antimicrobial peptides, are key factors in controlling the intestinal micro biota composition. Deficiencies in these genes lead to changes in the composition of the gut micro biota, causing leakage of endotoxins into the circulation, and the development of low-grade chronic inflammation and insulin resistance. Dietary composition can also affect the micro biota: a diet rich in saturated fats allows the expansion of pathobionts that damage the intestinal epithelial cell layer and compromise its barrier function, so-called “leaky gut” [22,23]. In conclusion we can say that insulin resistance leads to a great number of comorbidities, which can be avoided or postponed if people would have a healthy weight and an active lifestyle.

Insulin resistance develops over the years, but can be seen in young and old people. Even 23% of people with a BMI <25 kg/m^2^ appeared to be insulin resistant [24]. The incidence of insulin resistance is 48.7% in overweight and 66.3% in obese patients [25]. In general, the longer the duration of the obesity and the higher the BMI, the more insulin resistant a patient will be, although genetic predisposition cannot be ruled out, considering the fact that obesity and type 2 diabetes are strongly hereditary conditions The use of anti-depressants and anti-psychotic drugs enhances insulin resistance [26,27]. A sedentary lifestyle also promotes developing insulin resistance and low free fat mass [14]. This phenomena causes the fatigue that many patients complain about. Psychosocial stress is strongly related to insulin resistance, which proved to be an independent predictor of waist and HOMA-IR only among participants with a low level of spirituality [28].

## 3. Metabolic Syndrome

Most health care professionals are familiar with metabolic syndrome. The criteria for metabolic syndrome can vary slightly per country, but, in general, are characterized by: blood pressure >130/>85 mm/Hg; elevated triglyceride level: >1.7 mmol/L; decreased HDL <1.03 mmol/L(men); <1.29 mmol/L (women); and a large waist circumference: >102 cm (men); >88 cm (women); IFG >6.1 mmol/L [27,28]. In fact, the criteria of metabolic syndrome describe the changes due to insulin resistance, and it is fair to assume that insulin resistance is the cause of metabolic syndrome. It would, therefore, be more just to use the term insulin resistance or insulin resistance syndrome (IRS) instead of metabolic syndrome, in order to describe the condition by its cause rather than by its outcomes, which seem a bit arbitrary considering the abundance of abnormal physical parameters that are connected to IRS. Insulin resistance in primary care or clinical situations can be calculated through the homeostasis model assessment (HOMA-IR) method, or fasting insulin can be measured in blood samples, although none of these methods are common practice in primary care or in clinics for obese patients with comorbidities.

## 4. Treatment

Weight loss through diet and exercise leads to significant health improvement and is, therefore, the key in treatment of obesity and insulin resistance related comorbidities [29,30,31,32,33,34]. Early recognition of overweight or a large waist circumference when patients visit a physician with vague complaints about their health, or when comorbidities are found, is crucial. This is the moment to discuss lifestyle, stress, sleep, and eating habits. This should also be the moment to establish whether a patient is aware of the influence he has on his own health, thus, promoting self-management. The earlier the weight problem, or more specifically the visceral fat problem, is addressed, the greater the chance that weight loss is possible, that elevated blood parameters will improve without medication, and health can be restored [35,36].

Many physicians hesitate to discuss lifestyle with their patients, reluctant to interfere in their lives. However, we have no choice. Patients partly develop overweight because of the obesogenic environment, where physical exercise is not natural, where people have to work long hours, unhealthy food is everywhere and cheap, traditional meal patterns disappear, and where cooking skills have diminished to very simple dishes. In addition, more recent evidence shows that caloric restriction and exercise are potent interventions to promote adipose tissue weight loss and alteration of immune cell phenotype [37,38,39,40].

Patients need help in this complicated situation. They need to be referred to health professionals, more specifically dietitians, for advice and guidance, rather than to commercial programs. Another problem is that we do not identify is patients with insulin resistance as such and assume that all patients benefit from a diet based on national dietary guidelines. A constant high level of serum insulin, in fact, may cause weight gain with normal quantities of carbohydrates, leaving the patient frustrated that weight loss for her or him is impossible [41]. Even systematic reviews make no distinction between patients that are strongly insulin resistant and those who are not, stating that choice of diet makes no difference [42]. The Mediterranean diet and the DASH diet are evidence-based diets that are beneficial for weight loss and improvement of comorbidities, e.g., glucose levels, lipids, cardiovascular parameters, and insulin sensitivity [43,44,45,46]. Both diets are rich in whole grains, vegetables, fruits, legumes, nuts, fish, and mono unsaturated fat, and low in alcohol, red meat, sugar containing beverages, refined carbohydrates, and saturated fat. The DASH diet also promotes the use of sufficient quantities of low-fat dairy products. However, these diets need to be administered and defined carefully where the carbohydrate content is concerned, because a popular interpretation of the Mediterranean diet is that an abundance of low fiber products such as pasta, rice, and quinoa are part of the scheme, which, on the contrary, are harmful in case of severe insulin resistance. Low-carbohydrate (sometimes called ketogenic diets), low-GI, Mediterranean, and high-protein diets lead to a greater improvement in glycemic control compared to control diets. Low-carbohydrate, high protein and Mediterranean diets lead to greater weight loss than controls [44]. The composition of the diet in terms of macro nutrients can make a difference: isocaloric diets rich in MUFA compared to diets rich in carbohydrates and fiber, gave a stronger reduction of liver fat, regardless of exercise [47]. The patient is best helped with a tailor-made dietary advice with a lowered level of carbohydrates [48], preferably with a low glycemic index [49]. The amount of carbohydrates should be based upon thorough assessment of the patient in terms of history of previous weight loss attempts (how successful were they), BMI and waist circumference, medication, mental condition, positive family history of obesity and other comorbidities, sleep, and stress. Ideally speaking, patients are referred to the dietitian with a diagnosis of their fasting insulin level. Protein intake can be as high as 1.2 to 1.5 kg body weight, but not more than 100 g per day, evenly spread over three meals and a maximum of two in-between snacks, including 3 g of the essential amino acid leucine per meal [50]. Leucine is present in dairy products, which makes them essential to the diet. In this dietary advice micro nutrients are equally important: iodine, selenium, thiamine, riboflavin, magnesium, manganese, hydrocobalamin, folic acid, vitamin D, vitamin C, tocoferol, zinc, copper, and chromium [51,52,53,54]. The requirements for iodine, thiamine, magnesium, manganese, and selenium are not met when a low carbohydrate diet is prescribed. This implies that daily supplementation with one dose of the Advised Dietary Intake is necessary. A diet high in fiber supports the micro biota in the duodenum to produce short-chain fatty acids, thereby promoting energy expenditure and protecting against inflammation and insulin resistance. The interactions of the micro biota, innate immunity, and diet play an important role in controlling metabolic homeostasis. A properly functioning innate immune system, combined with a low-fat and high-fiber diet, is important in preventing dysbiosis and reducing the susceptibility of developing metabolic syndrome and its associated cardiovascular diseases [22]. A low fiber diet on the other hand enhances insulin resistance [55].

Furthermore, exercise is essential to tackle insulin resistance and to promote weight loss. Chronic exercise exerts potent anti-inflammatory effects [56,57,58], and these effects are likely mediated by direct effects on the immune system and a reduction in visceral fat, including diminished release of proinflammatory cytokines and chemokines from adipocytes [59]. Endurance exercise is associated with reduced induction of proinflammatory signaling and diet-induced obesity [60,61,62]. Furthermore, treadmill exercise reduces adipose tissue macrophage infiltration and promotes an anti-inflammatory immune cell phenotype [63]. During exercise, skeletal muscle is thought to produce and secrete a host of anti-inflammatory cytokines that are shown to experimentally alter immune cell function and phenotype. Walking, cycling, swimming are advised, one hour per day, but every other day in the first phase, to prevent over training, although good results have been reported with 150 min of moderately intense activity per week [34]. Patients with a very low fat free mass (measured by a four point body impedance) benefit from weight lifting and other muscle promoting exercises.

Finally, patients need help to start facing emotional barriers that prevent them from losing weight, or that may have caused the weight gain. We need to face the fact that many patients are part of an environment that is counterproductive for a healthy lifestyle and they need coaching to change things step-by-step. Insulin resistance can be cured, but it takes time. In most cases, therapy of less than a year will not lead to the desired result.

## 5. Conclusions

Treatment of insulin resistance syndrome (IRS) should start at the earliest stage possible, when comorbidities are still reversible, and includes personalized dietary advice and counseling, preferably by a dietitian, to tackle food habits and emotional barriers that prevent weight loss or cause relapse. An exercise program is an essential part of the treatment.
